# Adult isolated coronary artery ectasia: clinical features and long-term outcomes

**DOI:** 10.3389/fcvm.2026.1757073

**Published:** 2026-02-26

**Authors:** Yihan Weng, Jiquan Xiao, Xiang He, Yusi Huang, Wenzhi Hu, Renshang Xu, Huimin Yu

**Affiliations:** 1 Shantou University Medical College, Shantou, China; 2 Department of Cardiology, Guangdong Cardiovascular Institute, Guangdong Provincial People’s Hospital, Guangdong Academy of Medical Sciences, Southern Medical University, Guangzhou, China; 3 South China University of Technology, Guangzhou, China; 4 Guangdong Academy of Medical Sciences, Guangdong Cardiovascular Institute, Guangdong Provincial People’s Hospital, Guangzhou, China; 5 Southern Medical University, Guangzhou, China; 6 Department of Cardiology, Guangdong Provincial People’s Hospital’s Nanhai Hospital, Foshan, China

**Keywords:** adult, angiography, ischemia, isolated coronary artery ectasia, prognosis

## Abstract

**Background:**

Adult isolated coronary artery ectasia (ICAE) is a rare disease characterized by dilation of coronary arteries in the absence of significant stenosis. Its long-term prognosis and optimal management remain unclear. This study aimed to investigate the clinical and long-term outcomes of adult ICAE compared to controls with normal coronary arteries.

**Methods:**

This retrospective analysis utilized prospectively maintained coronary angiography databases at Guangdong Provincial People's Hospital (2012–2022). ICAE was defined as ≥1.5 times dilation with <20% stenosis. Adult patients meeting these criteria, after excluding cases with significant stenosis or secondary causes, were matched 1:1 by age and sex to controls with normal coronary arteries. Clinical, laboratory, ECG, echocardiographic, and angiographic data were collected. The primary outcome was all-cause mortality, and the secondary outcome was major adverse cardiovascular events (MACE).

**Results:**

The study included 171 adult ICAE patients and 171 matched controls. Compared to controls, ICAE patients exhibited a higher prevalence of hypertension, elevated cardiac biomarkers, and more frequent ECG abnormalities. Angiography showed a predilection for the LAD (70.8%) and frequent multivessel involvement; slow flow was noted in 26.9%. After a median 6.2-year follow-up, ICAE patients had a significantly increased risk of MACE (HR 2.17, 95% CI 1.23–3.82, *p* = 0.006), while all-cause mortality was similar (HR 1.07, 95% CI 0.43–2.63, *p* = 0.886).

**Conclusions:**

Adult ICAE exhibits distinct clinical and angiographic features, consistent with a chronic ischemia–like phenotype and possible association with elevated MACE risk.

## 1 Introduction

Coronary artery ectasia (CAE) is an uncommon abnormality of the coronary vasculature. It is typically defined as a dilatation of a coronary segment exceeding 1.5 times the diameter of an adjacent normal segment (1, 2). Detected in in approximately 3%–8% of patients undergoing invasive coronary angiography (ICA) (3). CAE most frequently coexists with atherosclerotic coronary artery disease (CAD) (4). Beyond atherosclerosis, other documented include etiologies, including Kawasaki disease, vasculitis, syphilis, obstructive sleep apnea, and iatrogenic injury (5, 6).

**Figure 1:**
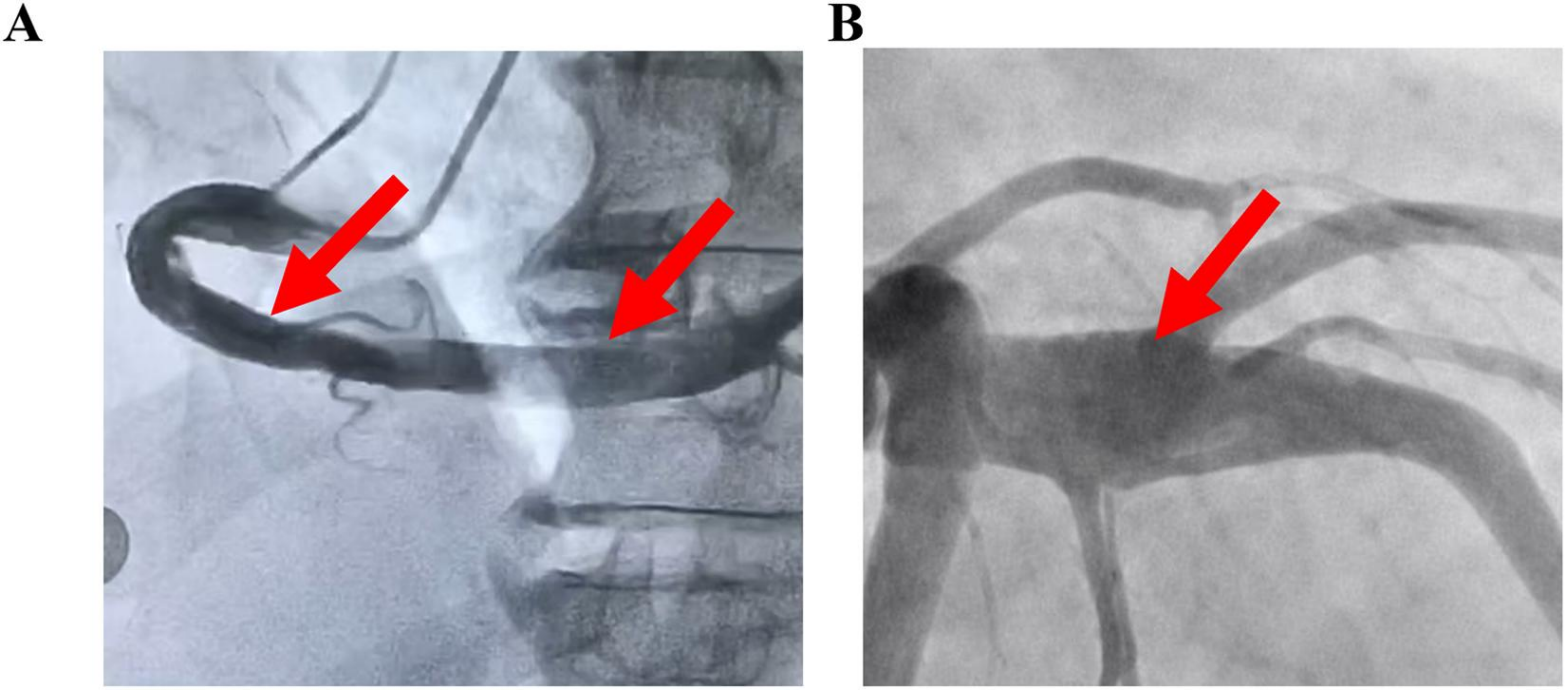
**(A)** Diffused ectasia of coronary artery. **(B)** Localized ectasia of coronary artery.

A rare subset of patients presents with ectasia in the absence of significant stenosis or secondary causes. This condition, termed isolated coronary artery ectasia (ICAE), is reported in fewer than 1% of angiographic series in adult (7, 8). Morphologically, ICAE manifests in two primary forms: diffused (Figure 1A) and localized or segmental ectasia (Figure 1B), and classified into four types, using the Markis classification: Type I (diffuse ectasia of two or more vessels), Type II (diffuse ectasia in one vessel with localized involvement in another), Type III (diffuse ectasia of a single vessel), and Type IV (localized or segmental ectasia only) (9).

Although adult ICAE appears to be an isolated structural anomaly, it may precipitate angina, and even myocardial infarction (MI) (5, 10). The proposed mechanisms for these complications include disturbed coronary flow, *in situ* thrombosis, and distal embolization (11). Despite these potential consequences, the natural history and optimal management of ICAE remain poorly defined (4, 9). Historically, the condition has been managed as a variant of atherosclerosis, leading to the empirical use of antiplatelet and lipid-lowering therapies (12). However, the evidence supporting these interventions is limited. As summarized in Table 1, most prior investigations into ICAE have been constrained by small sample sizes, heterogeneous diagnostic definitions, and relatively short follow-up durations (7, 9, 13–17). These limitations have led to inconsistent conclusions regarding the long-term prognosis of ICAE, particularly when compared to individuals with angiographically normal coronary arteries (18).

**Table 1:** Summary of previously published studies on ICAE.

First author and year	Sample size	Angiographic characteristics	Follow-up duration	Main clinical outcomes
Demopoulos, 1997 (7)	31	/	2.3 ± 1.1 years	4.8% UA; No MI, PCI or CV death
Zografos, 2013 (9)	35	LAD (48.6%); Diffuse ectasia (60%); Markis type IV (40%)	/	/
Zhang, 2015 (13)	76	RCA (64.5%); multivessel involvement (57.9%); Markis type I (48.7%)	Median 2.9 years	No difference in survival or event-free survival vs CAD + CAE
Malviya, 2017 (14)	52	Multivessel involvement (38.3%); Markis type IV (51.9%);	2.3 ± 1.7 years	Mostly benign course; MI 1.9%; UA 5.7%
Boles, 2019 (15)	41	/	Median 11.4 years	MACE 43.7% (*p* = 0.26); CV death 12%, higher than normal people (*p* = 0.03)
Willner, 2020 (16)	40	LAD (77.5%); multivessel involvement (42.5%)	6 ± 3.6 years	MACE 25%; no deaths
Yang, 2024 (17)	18	RCA (77.8%)	1 year	No difference in angina, MI, or CV death vs normal people

UA, unstable angina; MI, myocardial infarction; PCI, percutaneous coronary intervention; CV, cardiovascular; LAD, left anterior descending artery; RCA, right coronary artery; CAD, coronary artery disease; CAE, coronary artery ectasia; MACE, major adverse cardiac events.

Given these uncertainties, we sought to characterize the clinical features of adult ICAE, examine its association with cardiovascular risk factors, and evaluate its long-term outcomes compared with matched controls with normal coronary arteries.

## 2 Materials and methods

### 2.1 Study population

Data for this retrospective analysis were derived from comprehensive angiography databases at Guangdong Provincial People's Hospital. These databases utilize longitudinal follow-up protocols to ensure robust long-term monitoring following ICA. Adult patients (aged ≥18 years) who underwent angiography for suspected angina between January 1, 2012, and December 31, 2022, were screened for eligibility. CAE was defined as a coronary segment dilated to at least 1.5 times the diameter of an adjacent normal segment. Angiographic assessments were performed independently by two cardiovascular imaging specialists, each possessing over 10 years of experience in angiographic imaging. Patients were included in the ICAE cohort if they met the diagnostic criteria for ectasia and exhibited <20% stenosis across all coronary vessels. All patients underwent clinically indicated ICA, performed at the discretion of treating cardiologists. ICAE was diagnosed retrospectively based on angiographic findings and no invasive procedures were performed for research purposes.

Subsequently, to ensure a strict ICAE cohort, we applied a rigorous and clinically pragmatic exclusion strategy. Patients with any angiographic evidence of ≥20% coronary artery stenosis were excluded. Secondary causes of coronary ectasia were excluded primarily through detailed reviews of medical history, clinical presentation, and available medical records, including documented histories of Kawasaki disease, systemic vasculitis, syphilis, obstructive sleep apnea, or prior coronary intervention. Additional paraclinical investigations were not performed systematically in all patients but were obtained selectively when clinically indicated, in the presence of suggestive symptoms, abnormal findings, or at the request of patients (e.g., serology for vasculitis).

To minimize follow-up bias, we established a control group consisting of normal individuals from the same database (19, 20). For each patient in the ICAE group, a control was matched 1:1 based on gender, similar age (within one year difference), and ICA procedure timeframe (21).

### 2.2 Data collection

Baseline demographic and clinical data included age, sex, body mass index (BMI), smoking status, hypertension, and diabetes mellitus. Laboratory tests assessed: metabolic parameters: hemoglobin A1c (HbA1c), estimated glomerular filtration rate (eGFR); inflammatory markers: leukocyte count, high-sensitivity C-reactive protein (hs-CRP) (22); cardiac biomarkers: *α*-hydroxybutyrate dehydrogenase (*α*-HBDH), lactate dehydrogenase (LDH), creatine kinase (CK), creatine kinase-MB (CK-MB), high-sensitivity cardiac troponin T (hs-cTnT), and N-terminal pro–B-type natriuretic peptide (NT-proBNP) (13); and lipid profile: total cholesterol (TC), triglycerides (TG), high-density lipoprotein cholesterol (HDL-C), low-density lipoprotein cholesterol (LDL-C) (21).

Echocardiography was performed within one month of angiography to measure left ventricular ejection fraction (LVEF). Standard 12-lead electrocardiograms were reviewed for ST–T abnormalities and pathologic Q waves (23).

Angiographic data included the distribution of ectasia, number of affected vessels, Markis classification, and the presence of thrombus or slow coronary flow (9).

### 2.3 Outcomes and follow-up

The follow-up period spanned from the initial angiography until an outcome event or the study's conclusion. The primary outcome was all-cause mortality. The secondary outcome was major adverse cardiovascular events (MACE), a composite of sudden cardiac death, non-fatal myocardial infarction (MI), coronary revascularization, and hospitalization for unstable angina (UA) or heart failure (HF) (24). Mortality data were adjudicated using official death certificates and medical records. Cardiovascular events and hospitalizations were ascertained through clinic visits, telephone interviews, and review of hospital records (10, 25, 26).

### 2.4 Statistical analysis

Continuous normally distributed variables were expressed as mean ± standard deviation (SD) and compared using the Student's *t*-test. Non-normally distributed variables are presented as medians with interquartile ranges (IQR) and compared using the Mann–Whitney *U*-test. Categorical variables were expressed as frequencies and percentages and compared using the chi-square test. Survival curves were estimated using the Kaplan–Meier method and compared via the log-rank test. Hazard ratios (HR) and 95% confidence intervals (CI) were calculated using Cox proportional-hazards models. Due to the limited number of outcome events and the biological overlap between certain baseline variables and the ICAE phenotype, we prioritized unadjusted analyses to avoid overfitting and overadjustment bias. Statistical significance was defined as a *p*-value < 0.05. Analyses were conducted with SPSS software, version 27.0 (15, 17).

The study was conducted in compliance with the principles outlined in the Declaration of Helsinki. Ethical approval for this study was obtained from the Guangdong Provincial People's Hospital Ethics Committee. Since this retrospective study involved the review of existing medical records and did not involve intervention, the requirement for informed consent was waived by the ethics committee.

## 3 Results

### 3.1 Patient enrollment

**Figure 2:**
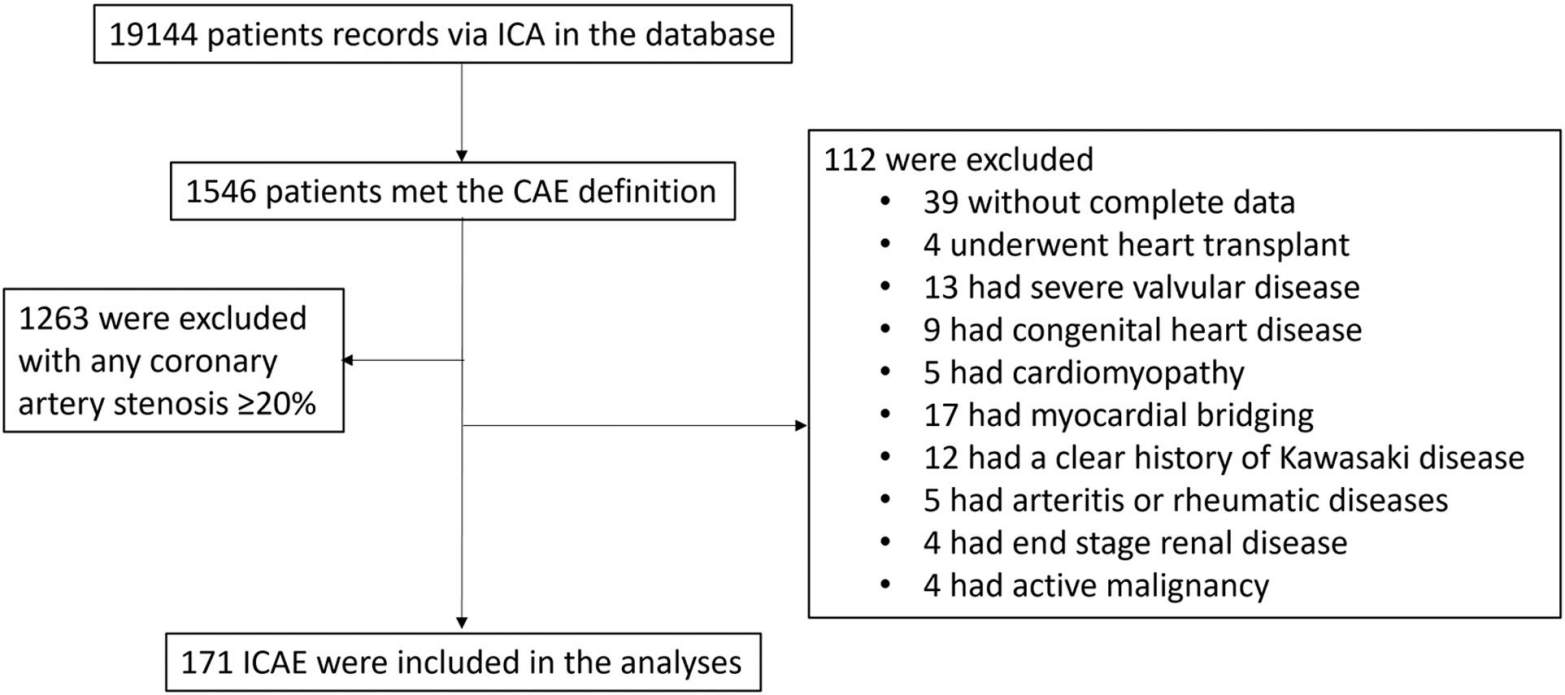
The enrollment process of ICAE patients.

The study population was derived from 19,144 ICA records in the database. As detailed in Figure 2, initial screening identified 1,546 patients who met the angiographic criteria for CAE. Subsequent application of exclusion criteria resulted in a final analytical cohort of 171 patients with ICAE. These exclusions included 1,263 patients with concomitant coronary artery stenosis of ≥20% and 112 patients with identifiable secondary etiologies for CAE or sever systemic disorders. For comparative analysis, a 1:1 matched control group (*n* = 171) consisting of patients with angiographically normal coronary arteries were selected from the same database.

### 3.2 Baseline characteristics

Baseline characteristics were well-matched for age (60.7 ± 12.0 years vs. 60.3 ± 11.7 years, *p* = 0.718) and sex (27.5% female in both groups, *p* = 1.000) (Table 2). BMI (24.6 ± 3.0 kg/m^2^ vs. 24.5 ± 3.0 kg/m^2^, *p* = 0.754) was also similar between the two groups, and the prevalence of diabetes mellitus (12.9% vs. 12.3%, *p* = 0.870) and current smoking (22.8% vs. 18.1%, *p* = 0.284) were comparable. However, ICAE patients exhibited a significantly higher prevalence of hypertension (56.7% vs. 37.4%, *p* = 0.001).

**Table 2:** Baseline characteristics of ICAE and control.

Variable	ICAE group (*n* = 171)	Control group (*n* = 171)	*p*-value
Age(years)	60.7 ± 12.0	60.3 ± 11.7	0.718
Female (*n*, %)	47 (27.5)	47 (27.5)	1.000
BMI (kg/m^2^)	24.6 ± 3.0	24.5 ± 3.0	0.754
Hypertension (*n*, %)	97 (56.7)	64 (37.4)	0.001
Diabetes (*n*, %)	22 (12.9)	21 (12.3)	0.870
Current smoking (*n*, %)	39 (22.8)	31 (18.1)	0.284
Leukocyte count (×10^9^/L)	7.1 ± 1.9	6.9 ± 2.0	0.379
hs-CRP (mg/L)	1.4 [0.4, 3.1]	0.7 [0.3, 3.7]	0.310
HbA1c (%)	6.0 ± 1.0	5.9 ± 0.9	0.559
eGFR (mL/min/1.73 m^2^)	81.5 ± 18.4	85.0 ± 18.4	0.057
Cardiac biomarkers			
*α*-HBDH (U/L)	124.9 ± 65.2	113.9 ± 35.7	0.067
AST(U/L)	26.6 ± 20.8	23.2 ± 9.1	0.061
LDH (U/L)	175.9 ± 57.6	162.7 ± 33.6	0.013
CK (U/L)	115.6 ± 82.5	110.6 ± 77.1	0.581
CK-MB (U/L)	11.3 ± 6.3	9.8 ± 4.3	0.016
hs-cTnT (pg/mL)	9.4 [7.1, 16.3]	7.5 [5.3, 9.4]	0.001
NT-proBNP (pg/mL)	69.2 [35.2, 217.1]	41.2 [19.5, 93.6]	0.001
Lipid profile			
TC (mg/dL)	174.1 ± 39.1	176.9 ± 37.9	0.500
TG (mg/dL)	140.3 ± 94.3	141.8 ± 78.8	0.874
HDL-C (mg/dL)	42.9 ± 11.1	40.8 ± 11.1	0.077
LDL-C (mg/dL)	106.8 ± 31.4	110.5 ± 31.5	0.279
ECG			
Pathologic Q-wave (*n*, %)	27 (15.8)	12 (7.0)	0.002
ST-T change (*n*, %)	78 (45.6)	46 (26.9)	0.001
LVEF (%)	63.7 ± 7.5	65.5 ± 6.2	0.027
Long-term medication^a^			
Antithrombotic (*n*, %)	97 (56.7)	27 (15.8)	0.001
Statins (*n*, %)	99 (57.9)	55 (32.2)	0.001
RAAS-i (*n*, %)	73 (42.7)	49 (28.7)	0.007
CCB (*n*, %)	52 (30.4)	32 (18.7)	0.012
Diuretic (*n*, %)	18 (10.5)	8 (4.7)	0.041

BMI, body mass index; hs-CRP, high-sensitivity C-reactive protein; HbA1c, hemoglobin A1c; eGFR, estimated glomerular filtration rate; alpha-HBDH, alpha-hydroxybutyrate dehydrogenase; LDH, lactate dehydrogenase; CK, creatine kinase; CK-MB, creatine kinase-MB; hs-cTnT, high-sensitivity cardiac troponin T; NT-proBNP, N-terminal pro b-type natriuretic peptide; TC, total cholesterol; TG, triglycerides; HDL-C, high-density lipoprotein cholesterol; LDL-C, low-density lipoprotein cholesterol; LVEF, left ventricular ejection fraction; ECG, electrocardiogram; RAAS-i, renin-angiotensin-aldosterone system inhibitors; CCB, calcium channel blockers.

^a^ Medication in this table refers to drugs prescribed for long-term use following patient discharge.

Laboratory analysis revealed several differences in key parameters between the groups (Table 2). Some cardiac biomarkers were significantly elevated in ICAE patients, including LDH (175.9 ± 57.6 U/L vs. 162.7 ± 33.6 U/L, *p* = 0.013), CK-MB (11.3 ± 6.3 U/L vs. 9.8 ± 4.3 U/L, *p* = 0.016), hs-cTnT [9.4 pg/mL [IQR 7.1–16.3] vs. 7.5 pg/mL [IQR 5.3–9.4], *p* = 0.001], and NT-proBNP [69.2 pg/mL [IQR 35.2–217.1] vs. 41.2 pg/mL [IQR 19.5–93.6], *p* = 0.001]. However, no significant differences were observed between the groups in inflammatory markers such as leukocyte count (7.1 ± 1.9 × 10⁹/L vs. 6.9 ± 2.0 × 10⁹/L, *p* = 0.379) and hs-CRP [1.0 mg/L [IQR 0.3–4.3] vs. 1.4 mg/L [IQR 0.4–3.2], *p* = 0.225], metabolic parameters such as HbA1c (6.0% ± 0.9% vs. 5.9% ± 0.9%, *p* = 0.559), eGFR (81.5 ± 18.4 mL/min/1.73 m^2^ vs. 85.0 ± 18.4 mL/min/1.73 m^2^, *p* = 0.057), and standard lipid profiles (TC, TG, HDL-C, LDL-C; all *p* ≥ 0.05).

ECG analysis also showed a higher prevalence of pathologic Q-waves (15.8% vs. 7.0%, *p* = 0.002) and ST-T changes (45.6% vs. 26.9%, *p* = 0.001) in the ICAE group (Table 2). LVEF was slightly lower in ICAE patients, although remaining within the normal range (63.7% ± 7.5% vs. 65.5% ± 6.2%, *p* = 0.021).

In addition, for long-term medications, ICAE patients exhibited significantly higher drug utilization, including renin-angiotensin-aldosterone system inhibitors (RAAS-i) (42.7% vs. 28.7%, *p* = 0.007), calcium channel blockers (CCB) (30.4% vs. 18.7%, *p* = 0.012), and diuretics (10.5% vs. 4.7%, *p* = 0.041). Remarkably, more than half of ICAE patients had taken antithrombotic therapy (56.7%) and statins (57.9%), whereas the control group was significantly lower at 15.8% and 32.2% (both *p* = 0.001).

### 3.3 Angiographic features of ICAE

Among ICAE patients, the left anterior descending artery (LAD) was the most commonly affected vessel (70.8%), followed by the left circumflex artery (LCX, 57.3%) and the right coronary artery (RCA, 56.1%). Left main (LM) involvement was observed in 18.7% of patients (Table 3). Regarding the number of affected vessels, 45.6% had single-vessel ectasia, while 19.9% and 34.5% had two and three affected vessels, respectively. Based on the Markis classification, Type IV was the most common (54.4%), followed by Type I (25.7%), Type II (11.1%), and Type III (8.8%). Additionally, slow blood flow was observed in 26.9% of patients, and coronary thrombus formation was rare (2.3%) (Table 3).

**Table 3:** Angiographic features of ICAE.

Angiographic features	*n* (%)
Ectatic coronary	
LM, *n* (%)	32 (18.7)
LAD, *n* (%)	121 (70.8)
LCX, *n* (%)	98 (57.3)
RCA, *n* (%)	96 (56.1)
Number of ectatic coronary	
1, *n* (%)	78 (45.6)
2, *n* (%)	34 (19.9)
3, *n* (%)	59 (34.5)
Markis classification	
Type I, *n* (%)	44 (25.7)
Type II, *n* (%)	19 (11.1)
Type III, *n* (%)	15 (8.8)
Type IV, *n* (%)	93 (54.4)
Slow blood flow, *n* (%)	46 (26.9)
Coronary thrombus, *n* (%)	4 (2.3)

LM, left man stem; LAD, left anterior descending artery; LCX, left circumflex; RCA, right coronary artery.

### 3.4 Long-term outcomes of the patients

#### 3.4.1 Primary outcome

**Figure 3:**
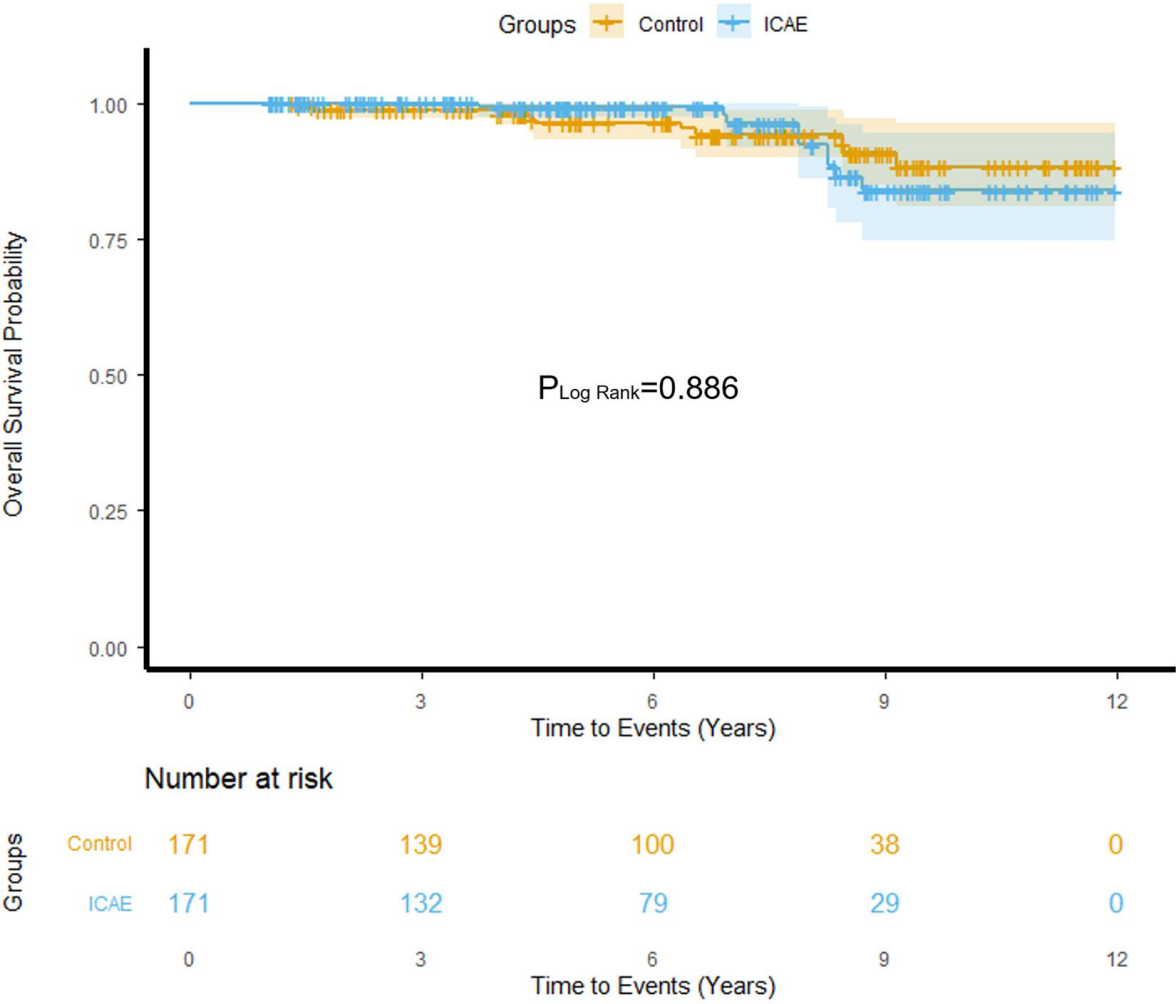
Overall survival probability of ICAE and control.

Patients were followed for a median of 6.2 years [IQR 3.6–8.8]. During follow-up, nine patients (5.3%) in the ICAE group and ten patients (5.9%) in the control group died. There was no statistically significant difference in all-cause mortality between the two groups (HR 1.07, 95% CI 0.43–2.63, *p* = 0.886) (Table 4). Kaplan–Meier survival analysis demonstrated no significant difference in overall survival between ICAE and control groups (Log-Rank *p* = 0.886) (Figure 3).

**Table 4:** Long term outcomes in ICAE and control.

Outcomes	ICAE group	Control group	HR (95% CI)	*p*-value
Mortality	9 (5.3%)	10 (5.9%)	1.07 (0.43–2.63)	0.886
Cardiovascular	5 (2.9%)	4 (2.3%)	1.51 (0.41–5.63)	0.539
Non-cardiovascular	4 (2.3%)	6 (3.5%)	0.78 (0.22–2.76)	0.698
MACE	33 (19.3%)	19 (11.1%)	2.17 (1.23–3.82)	0.006
Sudden cardiac death	2 (1.2%)	2 (1.2%)	1.45 (0.20–10.32)	0.709
Non-fatal MI	2 (1.2%)	1 (0.6%)	2.86 (0.26–31.96)	0.394
Coronary revascularization	3 (1.8%)	2 (1.2%)	2.25 (0.37–13.49)	0.376
Hospitalized for UA/HF	18 (10.5%)	9 (5.3%)	2.40 (1.07–5.34)	0.033
Non-fatal ischemic stroke	8 (4.7%)	5 (2.9%)	2.85 (0.25–31.92)	0.395

MACE, major adverse cardiovascular events; MI, myocardial infarction; UA, unstable angina; AHF, acute heart failure.

Regarding the causes of death, cardiovascular death occurred in five patients (2.9%) in the ICAE group and four patients (2.3%) in the control group. There was also no statistically significant difference in cardiovascular mortality between the two groups (HR 1.51, 95% CI 0.41–5.63, *p* = 0.539) (Table 4).

#### 3.4.2 Secondary outcome

**Figure 4:**
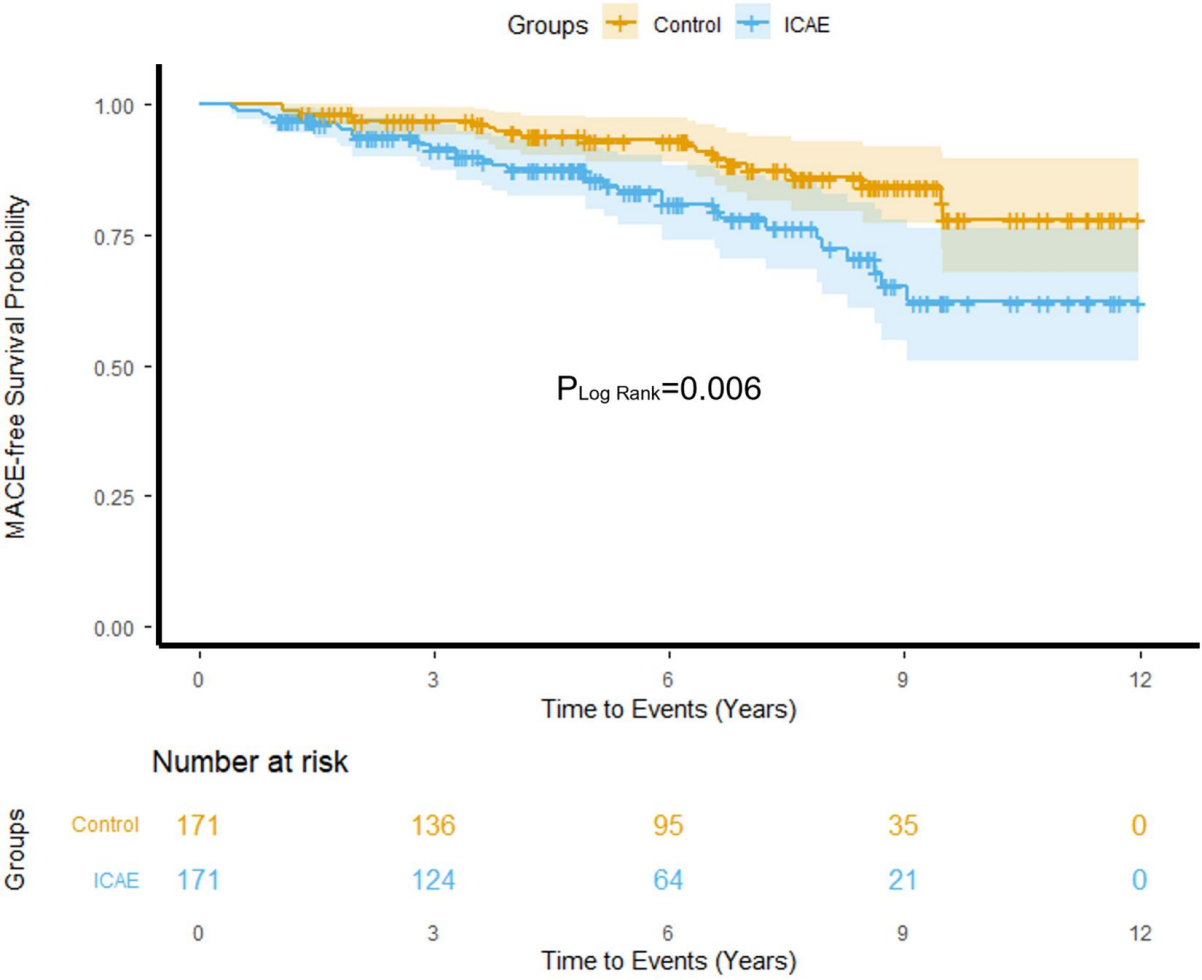
MACE-free survival probability of ICAE and control.

During follow-up, 33 (19.3%) patients in the ICAE group experienced MACE, compared to 19 (11.1%) in the control group. MACE occurred significantly more often in the ICAE group (HR 2.17, 95% CI 1.23–3.82, *p* = 0.006) (Table 4), with Kaplan–Meier analysis showing lower MACE-free survival in the ICAE group (Log-Rank *p* = 0.006) (Figure 4).

The increased risk of MACE in ICAE patients was primarily driven by hospitalization for UA or HF (10.5% vs. 5.3%; HR 2.40, 95% CI 1.07–5.34, *p* = 0.033), whereas hard endpoints, including: sudden cardiac death (1.2% vs. 1.2%; HR 1.45, 95% CI 0.20–10.32, *p* = 0.709), non-fatal MI (1.2% vs. 0.6%; HR 2.86, 95% CI 0.26–31.96, *p* = 0.394), coronary revascularization (1.8% vs. 1.2%; HR 2.25, 95% CI 0.37–13.49, *p* = 0.376), did not differ significantly between groups (Table 4).

## 4 Discussions

To our knowledge, this study represents the largest investigation specifically on long-term outcomes of adult ICAE to date. Our findings provide valuable insights into the clinical, angiographic, and long-term prognosis of this rare disease. Compared with a matched control group, ICAE patients exhibited a higher prevalence of hypertension and elevated cardiac biomarkers. Angiographic analysis revealed a predilection for the LAD but frequent multivessel involvement. Importantly, ICAE was associated with a significantly increased incidence of MACE, particularly UA/HF, while all-cause mortality remained comparable between the groups.

The pathogenesis of adult ICAE appears complex and multifactorial, with hypertension identified as a significant contributing factor. A recent meta-analysis included 40 study found an odds ratio of 1.44 for the association between hypertension and CAE (27). Sustained increases in intraluminal pressure may induce endothelial injury, triggering vascular remodeling and progressive arterial dilation (28, 29). This remodeling, driven by chronic hemodynamic stress, may manifest as progressive arterial dilation formation. This mechanism mirrors the formation of intracranial arterial dolichoectasia and aorta dilatation, where hemodynamic stress plays a central role (30, 31). However, other traditional risk factors for coronary atherosclerosis, such as obesity, smoking and diabetes (32–34), were comparable between the two groups in our study. These findings, coupled with the observation of smooth coronary lumens on angiography, suggest that traditional atherosclerosis is not the primary driver of ICAE and that the condition should likely be categorized separately from typical atherosclerotic disease (35). Similarly, our study also found no significant differences in standard lipid levels such as LDL-C and TG between ICAE patients and controls. However, emerging evidence points to alternative lipid-related mechanisms in ICAE pathogenesis. For example, Boles et al. identified disturbances in phosphatidylcholine and sphingomyelin levels (36), suggesting disruptions in fatty acid metabolism and increased susceptibility to premature apoptosis.

Inflammation is another proposed factor in ICAE development, affecting vessel wall structure and function. While previous studies have demonstrated elevated inflammatory markers like interleukins, tumor necrosis factor (TNF) and endothelial activation (1, 37) in ICAE, our study found similar leukocyte count and hs-CRP levels between groups. This discrepancy may reflect a lack of sensitivity in these general markers for the specific inflammatory pathways involved in ICAE, or it may be a result of the timing of measurement. Nonetheless, structural vascular changes mediated by inflammation likely contribute to the progression of disease, even in the absence of a high systemic inflammatory load (22, 38). Further research is needed to identify more specific inflammatory biomarkers and clarify inflammation's precise role in this rare disease.

A striking finding is that ICAE patients exhibited higher levels of cardiac biomarker, together with ECG abnormalities, which are commonly associated with myocardial ischemia or injury (39). Although LVEF remained within the normal range, its modest but consistent reduction in ICAE patients may reflect early or subclinical myocardial dysfunction. Unlike CAE with obstructive CAD, where slow flow and thrombus formation can precipitate MI through distal embolization or vessel occlusion (5), ICAE in the present study was characterized by a distinct clinical profile, with excess risk driven predominantly by UA/HF rather than MI or cardiovascular death. Similar patterns have been described in smaller cohorts reported by Malviya et al. and Willner et al. (14, 16). However, our study extends these observations by applying a stricter definition of isolated disease, enrolling a larger population, and providing longer-term follow-up. Collectively, these findings are consistent with the hypothesis that ICAE produces abnormal flow and a chronic ischemia-like state. It is insufficient to cause extensive myocardial necrosis but may provoke recurrent anginal symptoms and progressive myocardial stress, thereby contributing to the observed increase in non-fatal cardiovascular events (39). However, this interpretation is based on indirect clinical evidence, as direct functional assessments of ischemia (e.g., FFR) were not performed (40).

However, despite the associated cardiovascular risk, current clinical guidelines for CAD (34, 41) offer no specific recommendations for the management of adult CAE, particularly those with ICAE. This lack of guidance underscores the urgent need for evidence-based management strategies, as many adult ICAE patients are empirically treated with antiplatelet and lipid-lowering agents as primary or secondary prevention without clear evidence of benefit (42, 43). The higher rate of MACE in ICAE patients emphasizes the clinical concern regarding cardiovascular risk and reinforces the critical need for further research. The comparable all-cause mortality, despite the elevated incidence of MACE, warrants the need for extended follow-up to clarify the long-term prognostic implications of ICAE.

### Limitations

First, its retrospective, single-center design may limit the generalizability of the findings and the ability to draw definitive causal inferences. Although we applied strict angiographic definitions and excluded overt secondary causes, unrecognized subclinical etiologies cannot be entirely ruled out. Second, although multiple clinical, laboratory, and imaging abnormalities were observed, their limited sensitivity and specificity preclude their use as standalone diagnostic markers, and ICA remains the reference standard for diagnosis.

Third, we did not perform formal multivariable adjustment. Some candidate variables, such as hypertension, are biologically intertwined with the ICAE phenotype. These might represent upstream contributors or downstream consequences of the disease rather than independent confounders, making adjustment potentially prone to overadjustment bias. In addition, many disease-specific characteristics and treatment variables were present almost exclusively in ICAE patients, limiting the interpretability of joint multivariable modeling with control groups. Furthermore, the relatively small number of outcome events constrained the feasibility of stable multivariable models (44). Finally, pharmacologic therapy was not randomized or standardized; it was initiated or adjusted based on clinical judgment. Consequently, this likely reflects confounding by indication, whereby patients with more severe disease tend to receive more intensive therapy (9). Therefore, the independent effects of specific medical treatments on long-term outcomes might not be reliably assessed in this study.

## Conclusion

Our findings contribute to the understanding of adult ICAE by describing clinical characteristics and suggesting possible long-term prognostic implications. The condition appears to be characterized by abnormal coronary flow dynamics and a chronic ischemia–like phenotype, raising the possibility that ICAE may represent a distinct clinical entity rather than classic obstructive CAD. However, the observed association with an elevated risk of MACE, together with the absence of evidence-based management guidelines, points to an important gap in cardiovascular care. Further prospective studies will be valuable in clarifying optimal diagnostic and therapeutic strategies for this rare condition.

## Data Availability

The original contributions presented in the study are included in the article/Supplementary Material, further inquiries can be directed to the corresponding author.
